# Impact of Dental Health Education on “Specific Learning Needs” Children

**DOI:** 10.5005/jp-journals-10005-1329

**Published:** 2016-04-22

**Authors:** Aarti H Relwani, Shital Kiran, Rohan Bhatt, Megha Patel

**Affiliations:** 1 Consultant, Department of Pediatric Dentistry, PP Savani Multispeciality Hospital, Surat, Gujarat, India; 2 Professor and Head, Department of Pedodontics and Preventive Dentistry, Karnavati School of Dentistry, Gandhinagar, Gujarat, India; 3 Reader, Department of Pedodontics and Preventive Dentistry, Karnavati School of Dentistry, Gandhinagar, Gujarat, India; 4 Reader, Department of Pedodontics and Preventive Dentistry, Karnavati School of Dentistry, Gandhinagar, Gujarat, India

**Keywords:** Oral health education, Schoolteacher, Specific learning disability.

## Abstract

**Introduction:** This article compares and evaluates the effect of dental health education through schoolteachers and dental health professionals to “specific learning needs” children attending special school.

**Materials and methods:** A total of 71 “specific learning needs” children attending special school participated in the study. The baseline oral hygiene index-simplified (OHI-S) for all the participants was recorded. The training of schoolteachers was done using audiovisual and verbal methods on dental health facts and how to provide instructions on oral hygiene measures for reinforcing to the students. The students were randomly divided into three groups: Group 1 – No further dental health education by the schoolteachers or by the dental professionals was given to these students after the initial oral health education. Group 2 – In this group, the trained teachers taught students about the importance of oral health and demonstrated them brushing technique at intervals of 15 days, 1 month and 3 months. Group 3 – The dental professionals imparted dental health education and also demonstrated brushing techniques to these students at intervals of 15 days, 1 month and 3 months. Six months following the intervention a second examination was done to find out the OHI-S scores. Data analysis were done with Statistical Packages for the Social Sciences (SPSS) version 16 using one-way analysis of variance (ANOVA) statistical test.

**Results:** Group 2 demonstrated significant decline in OHI-S scores after intervention and all the three groups showed a statistically significant difference between the baseline OHI-S score and the scores after 6 months.

**Conclusion:** Schoolteachers can be utilized for reinforcing dental health education among “specific learning needs” children effectively.

**How to cite this article:** Relwani AH, Kiran S, Bhatt R, Patel M. Impact of Dental Health Education on “Specific Learning Needs” Children. Int J Clin Pediatr Dent 2016;9(1):31-34.

## INTRODUCTION

For years, children who had consistent difficulty in achieving a level of academic performance concomitant with their intellectual capacity were unfortunately labeled as retarded. Today the term learning disabled is applied to children who exhibit a disorder in one or more basic psychological processes, involving understanding or using spoken or written language. Learning disabilities affect between 3 and 15% of the population, which occur four times more frequently among boys than among girls.

Learning disabilities may be manifested in disorders in the form of listening, thinking, talking, reading, writing, spelling or arithmetic. These include conditions that have been referred as perceptual handicaps, brain injury, minimal brain dysfunction, dyslexia and developmental aphasia.^1^

Specific learning disability (SLD) means a disorder in which one or more of the basic psychological processes involved in understanding or using language, spoken and written, may manifest itself as imperfect ability to listen, think, speak, read, write, spell or to do mathematical calculations, including conditions, such as perceptual disabilities, brain injury, minimal brain dysfunction, dyslexia and developmental aphasia. The term does not include learning problems that are primarily the result of visual, hearing or motor disabilities, of mental retardation, emotional disturbance or environmental, cultural and economic disadvantage.

Specific learning disability usually manifests as persistent difficulty in learning to efficiently read (dyslexia), write (dysgraphia) or perform mathematical calculations (dyscalculia) despite normal intelligence, conventional schooling, intact hearing and vision, adequate motivation and sociocultural opportunity. Specific learning disability is presumed to be due to central nervous system dysfunction. Dyslexia affects 80% of all those identified as learning-disabled.^2^ The incidence of dyslexia in primary school children in India has been reported to be 2-18%, of dysgraphia 14% and of dyscalculia 5.5%.^3^

Oral health is a vital component of overall health, which contributes to each individual’s well-being and quality of life. It is an important aspect of health for all children and is more important for children with special health needs. Because oral hygiene affects one’s esthetics and communication, it has strong biological, psychological and social projections. People with disabilities deserve the same opportunities for oral health and hygiene as those who are healthy. Unfortunately, oral health care is one of the greatest unattended health needs in disabled people.^4^ Inability to maintain proper oral hygiene is one of the primary factors influencing the prevalence of dental disease in special learning needs children. The removal of plaque from teeth is a skill that can be mastered only when an individual has the dexterity to manipulate a toothbrush and an understanding of the objectives of this activity.^5^

Medical literature has shown that challenges to oral health are more complex for disabled children, who are often unable to adequately apply the techniques necessary to control plaque. In many instances, a disabled child’s oral hygiene care becomes the responsibility of another person, generally a parent or guardian, many of whom are emotionally or intellectually incapable of dealing with the health problems of their less fortunate affiliates.^4^ Evidence confirms that uptake of screening services for people with learning disabilities is lower and that they lack in oral health when compared with the general population.^6^

Educating and motivating patients to carry out effective daily oral hygiene can be challenging but immensely rewarding when efforts are successful. Children with SLDs have difficulty in managing their oral hygiene because of lack of cognition to understand and remember what needs to be done.^7^

The deficiency of organized dental health education programs for special schools for disabled and the paucity of published literature that demonstrates the effectiveness of oral health promotion programs prompted us to assess the practicability of utilizing the services of schoolteachers in the promotion of oral hygiene among “specific learning needs” children. The current study was, therefore, conducted to evaluate and compare the effectiveness of dental health education offered by schoolteachers with that offered by dental professionals among “specific learning needs” children.

## MATERIALS AND METHODS

A total of 71 students in the age group of 6-15 years attending a special education school participated in the study. Informed consent was obtained from all the participants’ parents, who were provided with detailed information on the study protocol. The ethical clearance to conduct this study was obtained from the Institutional Ethical Committee. Prior to any form of intervention, the baseline oral hygiene status of all students was assessed using the oral hygiene index-simplified (OHI-S) and its modification for deciduous dentition. An autoclaved set of instruments was used to record OHI-S of the students.

The teachers were trained using audiovisual and verbal methods on dental health facts and how to perform oral hygiene measure instructions for reinforcing to the students. Oral health education was imparted to all the students using audiovisual and verbal methods. Two well-trained and calibrated dentists conducted the preliminary examination to determine the level of oral hygiene among the students. The calibration of investigators in the application of OHI-S was done on 15 patients. An expert public health dentist explained to the investigators the method of examination, teeth to be examined and the criteria for scoring in the indices. A total of 15 selected students were examined by the first investigator and their OHI-S score was recorded on a data collection form. The other dentist completed the examination in the same manner. The scores given by the two investigators for oral hygiene for the same patients were compared to determine the interexaminer agreement, which was found to be 90%. The students were randomly divided into three different groups:

*Group 1:* No further dental health education by the schoolteachers or by the dental professionals was given to these students after the initial oral health education.

*Group 2:* In this group, the trained teachers reinforced students about the oral health importance and demonstrated them brushing technique at intervals of 15 days, 1 and 3 months.

*Group 3:* The dental professionals imparted dental health education and also demonstrated brushing techniques to these students at intervals of 15 days, 1 and 3 months.

The follow-up examination for the status of oral hygiene was done by the same investigators using the same protocol and data collection form at 6-month intervals.

At the follow-up examination, all the students were directed to come in their routine clothes and were pooled together in an auditorium hall. The students without any identification were examined by the investigators. This was done to avoid an investigator bias.

The change in the oral hygiene behavior of the students following dental health education was estimated after determining the difference in the oral hygiene status in the three groups. The mean OHI-S scores between the preliminary examination and the follow-up examinations in each group were compared using paired t-test and that between different groups using one-way analysis of variance (ANOVA). Statistical significance was fixed at 0.05.

## RESULTS

### Mean OHI-S Scores between the Three Groups at Baseline Examination

The mean baseline OHI-S score for the participants was 3.13 ± 0.82 (mean ± SD), suggesting a poor oral hygiene status. No statistically significant difference in the mean OHI-S score among the students in the three groups at baseline was found (OHI-S: p < 0.472, Table 1).

**Table 1:** **Table 1:** Baseline comparison of mean oral hygiene index-simplified scores between the three groups

		*Oral hygiene index-simplified*					
*Group code*		*Males (SD)*		*Females (SD)*		*Males and females* *combined (SD)*	
Group 1		3.19 (0.8)		3.2 (0.82)		3.19 (0.8)	
Group 2		3.06 (0.57)		3.12 (1.07)		3.09 (0.83)	
Group 3		3.12 (0.56)		3.1 (0.92)		3.11 (0.84)	
p-value		p < 0.254		p<0.911		p < 0.472	

SD: Standard deviation

### Mean OHI-S between the Three Groups, 6 Months following the Intervention

The mean OHI-S score for the sample in the second examination was 2.26 ± 1.39 (mean ± SD), suggesting that the status of oral hygiene was fair. Group 1 demonstrated highest OHI-S score (3.11 ± 0.95), which suggested poor oral hygiene, followed by group 3 (2.85 ± 0.97), suggesting fair oral hygiene. Group 2 demonstrated the lowest score (0.82 ± 0.41), suggesting good oral hygiene. There was a statistically significant difference in the mean OHI-S score among the students in the three groups (p < 0.0001, Table 2). Tukey’s *post hoc* comparison revealed a significant difference between group 1 and group 2, as well as between group 2 and group 3, with no significant difference between group 1 and group 3 (Table 2).

### Pre- and Postintervention Mean OHI-S in the Three Groups

There was a significant difference in the mean OHI-S in the follow-up examination in all the three groups. The participants in group 2 showed more improvement in the status of oral hygiene than those in the other two groups. The reduction in the mean OHI-S as compared to the baseline scores in this group was 2.27 (Table 3).

**Table 2:** **Table 2:** Comparison of mean oral hygiene index-simplified score between groups, 6 months following the intervention in the second and third groups but no intervention in the first group

		*Oral hygiene index-simplified*					
*Group code*		*Males (SD)*		*Females (SD)*		*Males and* *females* * combined (SD)*	
Group 1		3.05 (0.97)		3.15 (0.93)		3.11 (0.95)	
Group 2		0.86 (0.91)		0.78 (0.33)		0.82 (0.41)	
Group 3		2.55 (0.94)		3.15 (0.93)		2.85 (0.97)	
p-values		p < 0.0001		p < 0.0001		p < 0.0001	
Tukey’s *post hoc*		Group 1 *vs* group 2: p < 0.0001					
test for males and		Group 1 *vs* group 3: p < 0.752					
females		Group 2 *vs* group 3: p < 0.0001					

SD: Standard deviation

**Table 3:** **Table 3:** Comparison of preintervention and postintervention mean oral hygiene index-simplified scores in the three groups

		*Oral hygiene index-simplified*					
*Group* *code*		*Before* *intervention (SD)*		*After* *intervention (SD)*		*Statistical* *inference*	
Group 1		3.19 (0.8)		3.11 (0.95)		t = 2.672 df = 36 p < 0.011	
Group 2		3.09 (0.83)		0.82 (0.41)		t = 16.038 df = 39 p < 0.001	
Group 3		3.11 (0.84)		2.85 (0.97)		t = 3.717 df = 39 p < 0.001	
Overall		3.13 (0.82)		2.26 (1.39)		t = 9.302 df = 115 p < 0.001	

SD: Standard deviation

## DISCUSSION

Individuals with learning disabilities are perhaps the largest underserved population globally experiencing inequities in health access and outcomes.^8^ The oral health of people with learning disabilities is recognized as being poor compared with the general population.^9^ Research has identified that caries levels are sometimes higher in this group than in people without a disability.^10^ Additionally, people with learning disability have consistently been found to have poorer oral hygiene and greater periodontal needs than the general population.^11^

Oral health promotion is multidisciplinary, involving the local level like parents, schoolteachers and the community health care workers. India is a developing country with increasing population where a greater proportion of population is younger (in year 1980, approximately 320 million and in 2007 approximately 390 million). Since dental caries is the most prevalent disease of childhood, there is an urgent requirement to prevent our population from this risk. Since the dentist population ratio of our country is unfavorable, i.e., 1:47,000 (1:16,000 in urban areas and 1:32,000 in rural areas), dentists are unavailable in rural areas even for emergency dental services. The other avenues to deliver dental health education to the children appear to be through schoolteachers. Literature reveals that schoolteachers are the most competent and useful personnel other than dentists in providing dental health education to the schoolchildren.

The present investigation was carried out to determine and compare the effectiveness of oral health promotion among “specific learning needs” children through the dental team and the schoolteachers. The current study found improved oral hygiene status in the follow-up examinations of the three groups. The significant betterment of oral hygiene among students in the group reinforced by the schoolteachers clearly demonstrates that reiterative oral health education by schoolteachers brought about a coveted change in the oral hygiene status of these students. The personal communication by teachers might have indirectly motivated the students to perform better. The lack of difference between the other two groups with reference to oral hygiene status reveals that the infrequent dental health education, though offered by a dental professional, may not bring about a significant amount of change in the oral hygiene behavior observed when the same is delivered repeatedly by their teachers. Moreover, the fact that a teacher may evaluate the child’s performance and appreciate the child with best oral hygiene performance is in itself a motivation for the child to improve his/her oral hygiene practices.

Goel et al^12^ assessed the relative improvement in the knowledge achieved after imparting dental health education to school students of various socioeconomic groups, and the long-term effectiveness of conventional lecture technique revealed that the dental health education program was quite effective in improving the knowledge levels of most students. However, with the reversal of scores to preintervention levels after 1 year, the authors concluded that the single-lecture technique appears to be insufficient and it was important to reinforce knowledge in health education to bring about a long-term change in the oral hygiene practices. Our findings are in correspondence with the conclusion of the abovementioned study.

The results of our study correspond with the findings by Shenoy and Sequeira^13^ wherein they suggested that the reinforcement through repeated oral health education sessions, at 3-week intervals in the intervention schools, resulted in a considerable improvement in the knowledge of oral health, practices and reduction in the plaque index scores. The schools with frequent exposure to dental health education programs scored better in all aspects compared to schools with less frequent exposures. One study by Chachra et al^14^ found contradictory results to the current study that direct communication through dentist proved to be the most effective communication approach compared to indirect communication by schoolteachers and through members of social organizations for oral health promotion. These findings may be attributed to different methods of dental health education used in their study wherein pictorial story was used rather than visual demonstration of toothbrushing used in the current study.

## CONCLUSION

The idea of utilizing schoolteachers for repeated dental health education among “specific learning needs” children is definitely feasible as well as more effective than that by the professionals. Developing countries like India that lack organized school dental health programs, oral health policy and funds for such programs involving trained professionals can afford to train the schoolteachers. It is necessary for the public health authorities and health professionals to provide sustainable support to promote the effective utilization of schoolteachers for promotion of oral health in special schools for special children.
